# Smokeless tobacco use: pattern of use, knowledge and perceptions among rural Bangladeshi adolescents

**DOI:** 10.7717/peerj.5463

**Published:** 2018-08-21

**Authors:** Md Zahid Ullah, Jennifer NW Lim, Marie-Ann Ha, Md Mostafizur Rahman

**Affiliations:** 1 Faculty of Medical Science, Anglia Ruskin University, Cambridge, United Kingdom; 2 Faculty of Education, Work and Health, University of Wolverhampton, Wolverhampton, United Kingdom; 3 Department of Oral Surgery, Dhaka Dental College and Hospital, Dhaka, Bangladesh

**Keywords:** Smokeless tobacco, Adolescents, Rural Bangladesh, Health belief model, Social cognitive theory

## Abstract

**Background:**

The aim of the study was to investigate the practice and pattern of smokeless tobacco (SLT) use as well as the knowledge and perception about its ill effects among rural Bangladeshi adolescents.

**Methods:**

A cross-sectional survey was conducted among students aged 13–18 years in two rural secondary schools in Bangladesh in August 2015. Data were collected through a self-administered questionnaire which consists of topics derived from the Social Cognitive Theory and Health Belief Model (personal characteristics, environmental factors, self-efficacy, outcome expectancies, perceived susceptibility, perceived severity, perceived benefits, perceived barriers, and cues to action). Data analysis was performed using SPSS version 24. A descriptive analysis was conducted to determine the current pattern of SLT use and knowledge about its ill effects. A chi-square test and Fisher exact test were conducted to explore associations between variables. Lastly, a logistic regression model was used to locate the predictors for current SLT use.

**Results:**

A total of 790 students participated in the study. Among them, 9.5% (75) had used SLT at least once and 3.7% (29) were current SLT users. Males had a higher incidence of SLT use compared with females. The majority of students (77.3%) initiated SLT use between 10–13 years of age. ‘Zarda’ was the most common type of SLT used and most of the current users (86%) were able to buy SLT without age restrictions. Most of the current users (90%) wanted to quit SLT immediately; however, professional help was not available in schools. Overall, students had a good knowledge about the harmful effects of SLT with 54.8% (428) of respondents scoring in the good knowledge category. However, the majority of never SLT users (55.4%; 396) had a good knowledge compared to ever SLT users (42.7%; 32). Significant predictors of current SLT use included being a student aged 14 years and above (OR = 6.58, 95% CI [2.23–28.31]) as well as the variables of self-efficacy (OR = 5.78, 95% CI [1.46–19.65]), perceived barriers (OR = 0.30, 95% CI [0.10–0.74]), perceived benefit (OR = 0.21, 95% CI [0.05–1.03]) and perceived severity (OR = 0.36, 95% CI [0.16–0.91]).

**Discussion:**

This study demonstrates the need for comprehensive prevention and control programme in rural schools targeting young adolescents. Effective measure should be taken to reshape the attitude of rural adolescents towards self-confidence and competence, as to prevent SLT use.

## Introduction

Globally, more than 300 million people are currently using smokeless tobacco (SLT). Moreover, at least one in 10 adolescents aged 13 to 15 years use tobacco and this figure is much higher in low-income countries. A recent review of studies from 113 countries revealed that SLT use alone accounts for loss of over 6 million disability-adjusted-life-years (DALYs) and has caused 266,592 deaths from cancers and heart disease. Almost 85% of the global disease burden of SLT use is from the South Asia region, of which India accounts for 74% and Bangladesh accounts for 5% (Siddiqi et al., 2015).

SLT is used in a wide variety of forms in many countries of the world. Specifically, SLT is used orally by chewing, sucking, sniffing or applying it to the teeth or gums (toothpaste or powder) or inserting it in betel quid (National Cancer Institute and Centers for Disease Control and Prevention, 2014; IARC, 2007). The most popular forms of SLT products in South Asia are Zarda, Pan Masala, Khaini and Gul (Mutti et al., 2016). SLT products contain roughly 4,000 types of chemicals, including nicotine, carcinogens and other toxic chemicals, which are believed to cause negative health effects (Khariwala et al., 2013; Perfetti & Rodgman, 2012). Based on evidence from available epidemiological studies, the International Agency for Research on Cancer (IARC) concluded that SLT products, such as chewing tobacco, snuff or betel quid, are carcinogenic to humans (IARC, 2007).

Adolescents are at a stage of their life where they face different attractive choices of habits and these choices may change their future lifestyle (Ernst, Pine & Hardin, 2006). Using tobacco at an early age may lead to tobacco dependency, creating a strong addiction in early life and a difficult habit to quit (Kaduri et al., 2008). According to the WHO, three out of five adolescents who try any type of tobacco are more likely to become regular smokers in adulthood and consider other substance use (WHO, 1998). Regarding the long-term adverse effects of SLT use, adolescents are predisposed to a higher risk of oral cancer, oral mucosal lesions, periodontal diseases and heart disease in their middle age, which is also the most productive age of one’s life (National Cancer Institute and Centers for Disease Control and Prevention, 2014).

Several behavioural theories have been used to explain SLT initiation, continuation and cessation among adolescents, with social cognitive theory (SCT) and the health belief model (HBM) being the most widely used (Creamer et al., 2018; Cavazos-Rehg et al., 2016; Mantler, 2013; Nemeth et al., 2012). The SCT is one of the components of behaviourism, as it explains why an individual acquires and maintains certain behavioural patterns, such as SLT use (Bandura, 1986). In contrast, the HBM is a psychological model that attempts to explain and predict adolescents’ health behaviours by focusing on the attitudes and beliefs of an individual (Glanz, Rimer & Viswanath, 2015). Identification of social cognitive and HBM predictors of adolescent SLT use would be a primary step towards explaining this behaviour and for planning and developing SLT cessation interventions.

In Bangladesh, 57,000 people die and 1.2 million people suffer from tobacco-related diseases every year. More than 4.3 million people use different forms of tobacco and the health cost of tobacco-related diseases are double that of the revenue generated from this sector (WHO, 2015). SLT use contributed to a total of 320,000 DALYs lost and 13,329 deaths due to cancers and heart disease in Bangladesh alone (Siddiqi et al., 2015). Bangladeshi adolescents are facing a double challenge from smoking and SLT use, as SLT use is as prevalent as smoking tobacco in Bangladesh (National Cancer Institute and Centers for Disease Control and Prevention, 2014). Overall, 6.9% of Bangladeshi adolescents use any form of tobacco, but the exact number of adolescent SLT users is unknown (National Cancer Institute and Centers for Disease Control and Prevention, 2014; WHO, 2009). The recent Global Youth Tobacco Survey (GYTS) in Bangladesh reported that current SLT prevalence among school students aged 13 to 15 years was 4.5% and more prevalent among boys (5.9%) compared with girls (2.0%) (WHO, 2015).

The availability, affordability across regions and acceptance of tobacco in some cultures play vital roles in adolescent or youth tobacco use (Warren et al., 2009). The use of SLT products in rural areas has been deep-rooted in the Bangladeshi culture of hospitality (Mia et al., 2017). In Bangladesh, using SLT is regarded as a shared social activity that is performed with friends, relatives and family members and has even been integrated into social gatherings, such as festivals, weddings and religious gatherings (Sansone, 2014). In Bangladeshi society, younger people hesitate to smoke before their elders and they will never use or smoke cigarettes in front of their parents or seniors. However, SLT is an exception since chewing pan or betel leaf along with tobacco products is regarded as normal social behaviour and a symbol of hospitality in the rural areas of Bangladesh (Islam & AlKhateeb, 1995). Moreover, SLT use in Bangladesh is more prevalent among low socio-economic and socially disadvantaged populations (Mia et al., 2017; Huque et al., 2017; Azam et al., 2016). The high prevalence of SLT use in Bangladesh is associated with easy availability, low price and affordability, misconceptions regarding its useful health effects, lack of tobacco control regulations and weak enforcement of existing regulations (Huque et al., 2017).

The purpose of the study was to examine the current pattern and practice of SLT use among adolescents in a rural part of Bangladesh and their knowledge and perception about its ill effects. Possible predictors of current SLT habits were also explored to inform health promotion and preventive measures that could be targeted at this age group.

## Materials and Methods

This was a school-based cross-sectional study conducted in two rural secondary schools in Ramgati Upazila during October 2015. Ramgati Upazila is a subdivision of the Lakshmipur district in Bangladesh with a population of 261,002 with low socio-economic status (Bangladesh Bureau of Statistics, 2013). It is considered a rural community due to the dominant economic activity being farming. Secondary education in this rural area of Bangladesh is provided by 18 non-governmental schools serving approximately 8,994 students (Bangladesh Bureau of Statistics, 2013). The literacy rate in this area (39.3%) is lower than the national average (61.5%) (Bangladesh Bureau of Statistics, 2013).

Ethical approval for the present study was obtained from the Anglia Ruskin University Ethics committee (Ref: NS/jc/FMSFREP/15-039) and the head teachers of the participating schools in Ramgati Upazila. Informed consent was obtained from the students’ parents. Prior to the administration of the survey, the researcher (MZU) explained the study and the students were required to provide verbal consent to indicate voluntary participation. Students were informed of their right to decline participation or withdraw from the study at any time without consequence.

### Sample size and sampling technique

To reach a 95% confidence level with 2.5% marginal error and 10.1% expected ever SLT prevalence, a sample size of 553 was required (Daniel, 2009; WHO, 2015). Of the 18 secondary schools in Ramgati Upazila, with a total population of 8,994 students, 790 (8.78%) were recruited from two schools using a stratified method according to the enrolment size in each school. Initially the number of students for all 18 schools was collected from the local administrative office. From there, five schools with highest enrolment size were selected and from those five schools two schools were chosen randomly from their registration number.

### Survey tool and data collection

A self-administered questionnaire was adapted from the SLT module of the GYTS questionnaire version 1.00 (2012) (Global Youth Tobacco Survey Collaborative Group, 2012). Guided by the Social Cognitive Theory and Health Belief Model, the questionnaire was developed and comprised of closed-ended, open-ended and multiple response questions. Additional questions on types of SLT use, the reason for failing to quit SLT and eight knowledge questions were added from selected literature and validated surveys (Bhaskar et al., 2016; Abdullah et al., 2014; Kaduri et al., 2008).

The questionnaire was translated from English to Bengali and then back-translated to English by professional translators. It was also reviewed by the research team, local healthcare workers who had previously conducted a survey with students and teachers to ensure that the Bengali version was idiomatically appropriate for Bangladeshi adolescents. The questionnaire was piloted among five female and five male students from two secondary schools and was modified accordingly. Students took up to 15 min to complete the questionnaire.

### Study measures

The dependent measure or outcome variable for the present study was SLT use, which was classified into three main categories: ‘ever users’, ‘current users’ and ‘never users’ (WHO, 2015). Ever users were individuals who had used SLT at least once in their life, even if this was a tiny portion. Current SLT users were those who used SLT at least once in the last 30 days (WHO, 2015). Never users had never used SLT before. Additionally, respondents were asked, “What type or brand of SLT have you tried?”

### Social cognitive factors

“*Personal characteristics*” of the participants were measured by asking questions related to age, gender, father’s and mother’s profession, the age of initiation and SLT dependency. “*Environmental factors*” were assessed through querying about the source and access to SLT products. “*Self-efficacy*” was measured through respondent’s ability to deny SLT when offered by their friends. “*Outcome expectancies*” was measured by querying the reasons for SLT use.

### Health belief model

“*Perceived susceptibility*” was measured through students’ knowledge about harmful effects of SLT use and its contents, such as the development of white patches in the mouth, oral cancer, gum diseases and heart disease from SLT use. “*Perceived severity*” was assessed through asking respondents’ perception about the harmfulness of SLT compared to smoked tobacco. “*Perceived benefit*” was assessed by querying about the potential benefits of SLT use. “*Perceived barriers*” was measured by asking about the difficulty of quitting SLT. “*Cues to action*” was assessed by questioning about their experience receiving help to quit SLT and noticing the health warning on SLT packages.

### Data analysis

All the data were entered into MS Excel 2013 and exported into SPSS version 24 for analysis. Data analyses were conducted in multiple phases. In the first phase, a simple descriptive analysis (frequency, percentage, mean and median) was conducted to examine current SLT use behaviour as well as perception and knowledge about its ill effects. In the second phase, statistical analyses were performed to explore the association between different variables, such as socio-demographic variables, SLT use behaviours, perception and knowledge. Associations between categorical variables were assessed using a chi-square or Fisher exact test where appropriate. Knowledge was assessed using a scoring system where participants were given one point for each correctly answered question and zero otherwise. The sum of scores was calculated, and its relation to other variables was assessed using the student *t*-test or Mann–Whitney test when appropriate. The knowledge score was further categorised into poor knowledge (score of 0–2), average knowledge (score of 3–5), and good knowledge (score of 6–8). For the predictive model, the knowledge score was considered a nominal variable.

In the third phase, a multivariate logistic regression model was used to predict current SLT use. However, initially a univariate logistic regression was performed to determine variables that could be used to predict current SLT use. Akaike information criterion (AIC) was also extracted for each variable. Some variables were presented with different cut-off values. Each of them was assessed, and the variable with the lowest AIC was included in the final model. Only variables that were significant in the initial screening (univariate logistic regression) were included in the multivariate logistic regression. A correlation matrix was also constructed to ensure the absence of multicollinearity between variables included in the model. Exponentiated coefficients (odds ratio) were extracted from the model as well as the overall AIC of the model. Confidence intervals (95%) and Wald statistics were used to assess whether regression coefficients were significantly different from zero (null hypothesis). A receiver operator characteristics curve was plotted to assess whether the model could accurately classify the data. The Area Under the Curve (AUC) was 0.84 which is a good indicator of the model’s predictive power.

The odds ratio with a 95% CI was used to quantify the association. For the purpose of the analysis, a *P*-value of less than 0.05 was considered significant. Continuous variables were expressed as means or medians, whereas categorical variables were presented in frequencies or percentages. Data were missing for some of the questions; therefore, percentages were expressed according to the number of valid responses.

## Results

### Sample characteristics

Of the 820 enrolled students, 790 attended school on the day of the survey and all the students gave consent and participated in the study. More than half of the participants were male (63.3%). The mean age was 13.8 ± 0.07 years, and the median age was 14 years. There was no significant difference in the mean age of male (14.0 ± 0.07) and female students (13.7 ± 0.07). The predominant occupation of the participants’ fathers was farming (62.4%; 493), and almost all the participants’ mothers were housekeepers (98.9%; 781) (Table 1).

**Table 1:** Socio-demographic characteristics of the study participants.

Characteristics	Categories	Total % (*n*)	Ever SLT users	Non-users
Gender	Male	63.3% (500)	68.0% (51)	62.8% (449)
	Female	36.7% (290)	32.0% (24)	37.2% (266)
Age	13 or younger	40.4% (319)	16.0% (12)	42.9% (307)
	14	29.0% (229)	34.7% (26)	28.4% (203)
	15 or older	30.6% (242)	49.3% (37)	28.8% (205)
Father’s job	Farming	62.4% (493)	62.7% (47)	62.4% (446)
	Business	18.4% (145)	18.7% (14)	18.3% (131)
	Gov. employee	1.4% (11)	8.0% (6)	0.7% (5)
	Non-Gov. employee	6.1% (48)	4.0% (3)	6.3% (45)
	Doctor	1.3% (10)	1.3% (1)	1.3% (9)
	Teacher	3.0% (24)	2.7% (2)	3.1% (22)
	Daily labourer	1.9% (15)	0%	2.1% (15)
	Unemployed	0.4% (3)	1.3% (1)	0.3% (2)
	Others	5.2% (41)	1.3% (1)	5.6% (40)
Mother’s job	Housework	98.9% (781)	98.7% (74)	98.9% (707)
	Gov. employee	0.3% (2)	0%	0.3% (2)
	Teacher	0.9% (7)	1.3% (1)	0.8% (6)

**Notes.**

Others: Did not mention father’s profession.

SLT Smokeless tobacco

### SLT use behaviour

A total of 9.5% (75) reported ever using SLT. Approximately 3.7% (29) of the participants in this study or 38.7% of the ever users reported that they were currently using SLT. Males had a higher incidence of both ever and current SLT use with 68% (51) and 65.5% (19), respectively. Among the ever users, participants reported they started SLT use as early as the age of 7 years or younger (5.3%; four). However, most SLT users reported using SLT at the age of 12 or 13 years (34.7%; 25). Male SLT users (35.3%; 18) started using SLT at an earlier age (10–11 years) compared to female users (12–13 years) (41.7%; 10) (Table 2). Only two types of SLT were popular among the SLT users. Zarda was the most common type of SLT used (80%) followed by Pan Masala (20%) (Table 2). The majority used SLT less than once per day (65.5%; 19). Overall SLT dependency was low, where 89.3% (67) and 84% (63) of SLT users did not feel the need to use SLT first thing in the morning and did not feel a strong desire to use it again after using it once, respectively. Many respondents could not articulate their reason for using SLT (36.7%; 29). However, only male users (9.3%; five) and respondents aged 14 years and above (7.9%; five) used SLT because their friends also used. Regarding SLT cessation, 89.7% (26) of current SLT users wanted to quit, but none of them received help or advice from a program or professionals to do so. Regarding the attempt to quit SLT in the past 12 months, 34.5% (10) of current SLT users tried to stop SLT but failed. Between both genders, male students (42.1%; eight) had a higher rate of failure in quitting SLT compared with female students (20.0%; two) (Table 2).

Regarding access and availability, many students obtained SLT from social sources (41.4%; 12) and from stores and shops (34.4%; 10). Among current users who bought SLT products, 86% (12) were not refused because of their age. In terms of their exposure to the anti-tobacco messages, 45.3% (34) of respondents reported not seeing any health warning on SLT packages. In contrast, 29.3% (22) students had seen the health warnings and thought of quitting SLT.

The overall susceptibility to SLT use was 2.8% (22) when offered by friends and was significantly associated with ever SLT use. Specifically, 8% (six) of ever SLT users compared to 2.2% (16) of never users were likely to use SLT if offered by their friend (*p* = 0.01). The majority of students (59%; 466) thought it would not be difficult to quit once someone started using SLT and this was significantly associated with age. Specifically, 52.9% (128) of respondents 15 years or older believed it would be difficult to quit SLT compared to 35.8% (196) of respondents less than 15 years old (*p* < 0.001).

**Table 2:** Distribution of various factors across gender.

Measures	Categories	Male (*n*)	Female (*n*)	Total (*n*)	*P*-value
Ever SLT use	Never	62.8% (449)	37.2% (266)	100% (715)	0.38^a^
	Ever	68.0% (51)	32.0% (24)	100% (75)	
Current SLT use	No	63.2% (481)	36.8% (280)	100% (761)	0.84^a^
	Yes	65.5% (19)	34.5% (10)	100% (29)	
SLT type	Zarda	76.5% (39)	87.5% (21)	80.0% (60)	0.36^a^
	Panmasala	23.5% (12)	12.5% (3)	20.0% (15)	
Age of initiation	≤7	7.8% (4)	0.0%	5.3% (4)	**0.03^b^**
	8–9 years	3.9% (2)	8.3% (2)	5.3% (4)	
	10–11 years	35.3% (18)	25.0% (6)	32.0% (24)	
	12–13 years	31.4% (16)	41.7% (10)	34.7% (26)	
	14–15 years	21.6% (11)	25.0% (6)	22.7% (17)	
Number of days used	Did not use	62.7% (32)	58.3% (14)	61.3% (46)	0.28^b^
	1 to 2 days	27.5% (14)	16.7% (4)	24.0% (18)	
	3 to 5 days	5.9% (3)	20.8% (5)	10.7% (8)	
	20 to 29 days	0.0%	4.2% (1)	1.3% (1)	
	All 30 days	3.9% (2)	0.0%	2.7% (2)	
Frequency per day (current)	Less than once per day	63.2% (12)	70.0%(7)	65.5% (19)	0.71^a^
	At least Once per day	36.8% (7)	30.0% (3)	34.5% (10)	
Use SLT, first thing in the morning	No	90.2 % (46)	87.5%(21)	89.3%(67)	0.78^b^
	Yes sometimes	7.8% (4)	12.5% (3)	9.3% (7)	
	Yes always	2.0% (1)	0.0%	1.3% (1)	
Desire to use SLT again	Never	80.4% (41)	91.7% (22)	84.0% (63)	0.88^b^
	Within 60 minutes	9.8% (5)	8.3% (2)	9.3% (7)	
	1 to 2 hours	5.9% (3)	0.0%	4.0% (3)	
	1 to 3 days	2.0% (1)	0.0%	1.3% (1)	
	4 days or more	2.0% (1)	0.0%	1.3% (1)	
Reasons of using	Taste	13.0% (7)	12.0% (3)	12.7% (10)	0.47^b^
	Smell	5.6% (3)	4.0% (1)	5.1% (4)	
	Pleasure	27.8% (15)	28.0% (7)	26.7% (20)	
	Feels better	0.0%	4.0% (1)	1.3% (1)	
	Friend does	9.3% (5)	0.0%	6.3% (5)	
	Don’t know	37.0% (20)	36.0% (9)	36.7% (29)	
	Other reason	7.4% (4)	16.0% (4)	10.1% (8)	
Want to stop (Current)	Yes	94.7% (18)	80.0% (8)	89.7% (26)	0.58^b^
	No	5.3% (1)	20.0% (2)	10.3% (3)	
Tried to stop (Current)	Tried but unsuccessful	42.1% (8)	20.0% (2)	34.5% (10)	0.13^a^
	Did not try to quit	57.9% (11)	80.0% (8)	65.5% (19)	
Source of help to quit (Current)	A Program	0.0%	0.0%	0.0%	0.54^b^
	Friend	42.1% (8)	0.0%	27.6% (8)	
	Family	31.6% (6)	80% (8)	43.8% (14)	
	No help	26.3% (5)	20.0% (2)	24.1% (7)	
Source of SLT	School Shop	4.8% (1)	12.5% (1)	6.9% (2)	0.52^a^
	Street Vendor	14.3% (3)	0.0%	10.3% (3)	
	Someone else	38.1% (8)	50.0% (4)	41.4% (12)	
	Store near house	19.0% (4)	0.0%	13.8% (4)	
	Got it other way	23.8% (5)	25.0% (2)	24.1% (7)	
	On the way to school	0.0%	12.5% (1)	3.4% (1)	
Refuse to sell SLT	No	62.5% (10)	71.4% (5)	65.2% (15)	0.68^a^
	Yes	37.5% (6)	28.6% (2)	34.8% (8)	
Health Warnings	No	47.1% (24)	41.7% (10)	45.3% (34)	0.80^a^
	Yes, didn’t care	17.6% (9)	41.7% (10)	25.3% (19)	
	Yes, thought of quitting	35.3% (18)	16.7% (4)	29.3% (22)	
If offered by a Friend	No	97.8% (489)	96.2% (279)	97.2% (768)	0.26^a^
	Yes	2.2% (11)	3.8% (11)	2.8% (22)	
Difficult to quit	No	60.0% (300)	57.2% (166)	59.0% (466)	0.45^a^
	Yes	40.0% (200)	42.8% (124)	41.0% (324)	

**Notes.**

SLT Smokeless Tobacco

a Chi-Square Test.

b Fisher-Exact test

Significant results are bold.

### Knowledge about the harmful effects of SLT

To assess respondents’ knowledge about the adverse effects of SLT, students were asked eight questions. The overall knowledge about the harmful effects of smokeless tobacco use was good among respondents. The majority of adolescents (75.6%; 597) thought SLT use is bad for health. However, 21.2% (169) of study participants did not know whether SLT use is bad or good for the health. Over 32% (254) of students did not know whether SLT is less harmful compared to smoked tobacco (ST) and 25% (202) thought it is less harmful than ST. Additionally, 29.2% (231), 25.3% (200), 26% (206), and 25.4% (201) of students did not know SLT causes white patches in the mouth, oral cancer, gum diseases, and heart diseases respectively. Also, 32.5% (257) of respondents did not know SLT contains Nicotine.

The mean knowledge score was 5.19 ± 0.15. Male students (5.36 ± 0.09) were more likely to be knowledgeable compared to female students (4.9 ± 0.12). Students whose fathers’ profession was teaching (6.21 ± 0.37) had the highest mean score, and the lowest score was seen from students whose fathers’ occupation was farming (5.08 ± 0.09). Moreover, the average knowledge score was lowest among current SLT users (4.31 ± 0.40) followed by ever users (4.48 ± 0.20), and the highest average score was from the group who never tried SLT (5.27 ± 0.08).

Table 3 represents the knowledge category across various factors. Respondents were grouped into three main categories based on the total score distribution (poor knowledge, average knowledge, good knowledge). Overall, 54.2% (428) of respondents had good knowledge about the harmful effects of SLT. The majority of never SLT users (55.4%; 396) scored in the good knowledge category compared to ever users (42.7%; 32). Male respondents were more familiar with the SLT use hazards (56%) compared to females (51%) (Table 3). Knowledge was significantly associated with witnessing anti-tobacco messages on the SLT packages. Specifically, 61.8% (21) of ever SLT users who did not witness anti-tobacco messages on SLT packages had good knowledge compared to 26.8% who did see the anti-tobacco messages (*χ*2 = 9.87, *p* = 0.007, *φ* = 0.363). Knowledge was also significantly associated with respondent’s self-efficacy, where 55.2% (424) of respondents who would refuse to use SLT if offered by a friend had good knowledge compared to 18.2% (four) of those who would use SLT (*χ*2 = 11.98, *p* = 0.003, *φ* = 0.123). In addition, perceived barriers were significantly associated with knowledge, as 21.9% (102) of respondents who thought it would not be difficult to quit SLT once started had poor knowledge compared to 9.6% (31) of those who thought the opposite (*χ*2 = 20.95, *p* =  < 0.001, *φ* = 0.163) (Table 3).

**Table 3:** Distribution of knowledge index across various factors.

Measures	Category	Poor knowledge	Average knowledge	Good knowledge	*P*-value
Smokeless tobacco use status	Never users	15.9% (114)	28.7% (205)	55.4% (396)	0.05^a^
	Ever users	25.3% (19)	32.0% (24)	42.7% (32)
	Current use	27.6% (8)	24.1% (7)	48.3% (14)	0.28^a^
Gender	Male	14.6% (73)	29.4% (147)	56.0% (280)	0.08^a^
	Female	20.7% (60)	28.3% (82)	51.0% (148)
Age	14 < years	18.5% (59)	26.3% (84)	55.2% (176)	0.32^a^
	≥ 14 years	15.7% (74)	30.8% (145)	53.5% (252)
Father’s profession	Farmer	18.2% (93)	29.5% (151)	52.3% (267)	0.25^a^
	Other	14.3% (40)	28.0% (78)	57.7% (161)
Age of initiation	≤ 7 years	25.0% (1)	0.0%	75.0% (3)	0.17^a^^b^
	8–9	0.0%	0.0%	100.0% (4)
	10–11	20.8% (5)	33.3% (8)	45.8% (11)
	12–13	23.1% (6)	46.2% (12)	30.8% (8)
	14–15	41.2% (7)	23.5% (4)	35.3% (6)
Want to quit	Yes	30.2% (13)	34.9% (15)	34.9% (15)	0.54^a^
	No	12.5% (1)	37.5% (3)	50.0% (4)
Tried to quit last year	Yes	9.5% (2)	38.1% (8)	52.4% (11)	0.49^a^
	No	22.2% (12)	29.6% (16)	48.1% (26)
Health warning	Yes	29.3% (12)	43.9% (18)	26.8% (11)	**0.007^a^**
	No	20.6% (7)	17.6% (6)	61.8% (21)
If offered by friend	Yes	27.3% (6)	54.5% (12)	18.2% (4)	**0.003^a^**
	No	16.5% (127)	28.3% (217)	55.2% (424)
Difficult to quit	Yes	9.6% (31)	32.4% (105)	58.0% (188)	**<0.001^a^**
	No	21.9% (102)	26.6% (124)	51.5% (240)

**Notes.**

a Chi-Square Test.

b Fisher-Exact test.

Significant results are bold.

### Predictors of current SLT use

Initial univariate logistic regression analysis showed that age (*p* = 0.005), father’s profession (*p* = 0.046), self-efficacy (*p* = 0.001), perceived barriers (*p* = 0.029), perceived benefit (*p* = 0.014) and perceived severity (*p* = 0.008) were significant variables (Table 4). In the next stage, these variables were included in the multivariate logistic regression.

**Table 4:** Univariate regression analysis.

Labels	*P*-value	Odds ratio	AIC
Gender	0.800	0.904	252.532
Age (≥14 years)	**0.003**	6.154	239.14
Father’s profession	0.153	0.413	257.132
Farmer vs other (Other)	**0.046**	0.37	247.781
Mother’s profession	0.994	0	253.92
If someone offered you SLT, would you use it? (Yes)	**0.001**	6.604	245.285
Is it difficult to quit? (Yes)	**0.029**	0.363	247.001
Do you think SLT use is good for health?	0.106	6.875	252.882
Are there benefits of SLT to your body and health?	**0.014**	0.198	248.212
Does SLT cause less harm to your health compared to smoking tobacco?	**0.008**	0.268	243.674
Does SLT cause white patches in the mouth?	0.253	0.649	251.294
Can SLT cause oral cancer?	0.342	1.494	251.646
Does SLT cause gum diseases?	0.658	0.843	252.403
Does SLT cause heart diseases?	0.260	0.652	251.341
Does SLT contain nicotine?	0.079	0.509	249.454

**Notes.**

SLT Smokeless tobacco

Reference Group: Never used SLT, *AIC Akaike information criterion*.

Significant results are bold.

The multivariate regression analysis indicated that students who were 14 years old and older were 6.5 times more likely to be current SLT users compared to those younger than 14 years (*p* = 0.002) (Table 4). Perceived benefits and perceived severity of SLT use was associated with lower odds of current SLT use (OR = 0.21, 95% CI [0.059–1.03] and OR = 0.36, 95% CI [0.12–0.97], respectively) (Table 5). Self-efficacy was also a significant predictor of current SLT use where students who would use SLT if offered by a friend were more likely to be current SLT users compared to those who would not (OR = 5.78, 95% CI [1.46–19.65]). Lastly, perceived barriers regarding the difficulty of quitting SLT use was also a significant predictor of current SLT use where those who perceived quitting as easy were more likely to be current users compared to those who did not (OR = 0.30, 95% CI [0.10–0.74]) (Table 5). The model correctly classified 82% of the dependent variables.

**Table 5:** Multivariate analysis result.

Variables	Odds Ratio	SE	Wald statistic	*P*-value	95% CI for odds ratio	
					Lower	Upper
Intercept	0.083	0.07	−2.95	**0.003**	0.01	0.36
Age (≥14 years)	6.58	4.12	3.00	**0.002**	2.23	28.31
Are there benefits of smokeless tobacco to your body and health? (Yes) (Perceived benefit)	0.21	0.15	−2.17	**0.029**	0.05	1.03
Does smokeless tobacco cause less harm to health compare to smoking tobacco? (Yes) (Perceived severity)	0.36	0.185	−1.98	**0.046**	0.16	0.91
If someone offered you SLT, would you use it? (yes) (Self-efficacy)	5.78	3.75	2.70	**0.006**	1.46	19.65
Is it difficult to quit? (yes) (Perceived barrier)	0.30	0.14	−2.44	**0.014**	0.10	0.74
Father’s job (Other Job)	0.41	0.21	−1.701	0.08	0.13	1.05

**Notes.**

Reference Group: Never used SLT, CI, Confidence Interval; SE, Standard Error; SLT, Smokeless Tobacco.

Significant results are bold.

## Discussion

This is the first comprehensive study examining the current practice and pattern of SLT use and knowledge about the harmful effects among adolescents in the rural areas of Bangladesh. In our study, the current prevalence of SLT use was 3.7%, whereas existing GYTS, Bangladesh (2013) report showed that the national-level prevalence among adolescents was 4.5% (WHO, 2015). This declining trend could be explained by the recent implementation of the 15% Value Added Tax (VAT) and 30% supplementary duty on SLT products by the Bangladesh Government (Nargis, Hussain & Fong, 2014). The positive relation between the tax increase and decline of youth tobacco use was also reported in previous studies (Levy et al., 2017; Huang & Chaloupka, 2012). When compared with other South-East Asian countries, the current SLT prevalence in this study was higher than Indonesia, Sri Lanka and Thailand and Bhutan, and conversely lower than India, Nepal, Myanmar, Maldives and Timor-Thistle (National Cancer Institute and Centers for Disease Control and Prevention, 2014). The higher percentage of SLT use among boys in the present study showed similar trends to other South-East Asian countries: Bhutan (boys 27.2%; girls 19.8%), India (boys 11.1%; girls 6.0%) and Myanmar (boys 15.2%; girls 4.0%) (Sinha et al., 2014). Indeed, the cultural backdrop and symbols of maturity may play a key role in this trend (Hussain, Zaheer & Shafique, 2017).

The findings of the present study on early SLT initiation ties well with previous studies where adolescents started using SLT as early as 12 years old or younger (Liu et al., 2015; Muttapppallymyalil, Sreedharan & Divakaran, 2010; Kaduri et al., 2008). Initiating SLT at an early age may lead to tobacco dependency in the future (Haddock et al., 2001). This scenario stresses the need to extend the coverage of the Bangladesh anti-SLT campaign to primary school students (5th grade) rather than just focusing on secondary schools (6th grade onwards). Simultaneously, designing an effective adolescent SLT cessation campaign is challenging because adolescents have easy access to different forms of tobacco in the market as evidenced by the initiation of smoked tobacco being highest in the 12–13 years age group in the country (Arora et al., 2010). Therefore, a cessation programme that addresses the multiple forms of tobacco use in Bangladesh would be more effective (Sidhu et al., 2016). The necessity of a cessation programme was also stressed from another key finding of this study where the majority of current SLT users wanted to quit SLT but received no professional help. The lack of help from health promotion boards and health professionals was also reported by the WHO. In particular, only 8.9% students in Bangladesh had received help from a programme or professional to quit smoking (WHO, 2015). Much work is needed to increase the capacity to promote cessation of SLT in this population group.

Access and availability play a key role in influencing adolescent SLT use. Bangladesh has a law that bans selling SLT to and by minors (WHO, 2013). However, in line with the previous study, our study results showed the majority of students were able to buy SLT from stores without any restriction (Islam et al., 2016). Additionally, since May 2013, a new tobacco control amendment was put in place that requires graphic health warnings to be printed on tobacco (smoked / SLT) packages that cover at least 50% of the principal surface area (WHO, 2013). Nevertheless, our study findings demonstrated poor implementation of this law in the rural areas of Bangladesh.

Multivariate logistic regression analysis showed several constructs were strong predictors of current SLT use, namely, perceived benefit, perceived severity, self-efficacy and perceived barriers. These findings demonstrate the need for an effective anti-SLT campaign in rural areas of Bangladesh addressing the above factors to improve knowledge and perception of SLT use and its ill effects. Previous study results showed that health education campaigns based on the HBM and social cognitive factors were effective in improving knowledge and attitude about tobacco use hazards and enhancing success in quitting tobacco and preventing relapse (Elshatarat et al., 2016; Renuka & Pushpanjali, 2014).

We used a validated questionnaire from the WHO’s GYTS that enabled comparison of our results with other similar studies in the field. However, our study is not without limitations. Firstly, we did not have any information related to parental SLT use. In addition, the study was conducted in a rural area of Bangladesh and our results are therefore not generalisable to urban areas. Moreover, we collected self-reported data whereby both underreporting and over-reporting by the participants were plausible. Another limitation was not including adolescents who do not go to school, as they tend to have a higher risk of using tobacco and other substances (Kautilya, Sathish & Hegde, 2015).

## Conclusion

Our evidence suggests that SLT use in the rural areas of Bangladesh is low compared to other neighbouring countries. However, initiation of SLT at an early age is a public health concern. Lack of professional help to quit SLT and poor implementation of tobacco control laws were prevalent. Overall knowledge about SLT use and its ill effects was good, but this score was lower among SLT users compared to non-users. Professional help to quit SLT, tightening the tobacco control laws in rural areas and developing an educational health campaign focusing on young adolescents and different forms of tobacco use can reduce the current and future burden of adolescents SLT use in rural areas of Bangladesh.

##  Supplemental Information

10.7717/peerj.5463/supp-1 Dataset S1 School survey data

10.7717/peerj.5463/supp-2 Supplemental Information 1 Survey instrument Data collection Instrument

10.7717/peerj.5463/supp-3 Supplemental Information 2 Model validation

## Additional Information and Declarations

### Data Availability

The following information was supplied regarding data availability: The raw data are provided in a Supplemental File.

## References

[ref-1] Abdullah AS, Driezen P, Ruthbah HU, Nargis N, Quah A, Fong TG (2014). Patterns and predictors of smokeless tobacco use among adults in Bangladesh: findings from the International Tobacco Control (ITC) Bangladesh survey. PLOS ONE.

[ref-2] Arora M, Tewari A, Tripathy V, Nazar GP, Juneja NS, Ramakrishnan L, Reddy KS (2010). Community-based model for preventing tobacco use among disadvantaged adolescents in urban slums of India. Health Promotion International.

[ref-3] Azam NM, Shahjahan M, Yeasmin M, Ahmed UN (2016). Prevalence of smokeless tobacco among low socioeconomic populations: a cross-sectional analysis. PLOS ONE.

[ref-4] Bandura A (1986). Social foundations of thought and action: a social cognitive theory.

[ref-5] Bangladesh Bureau of Statistics (2013). District Statistics 2011 (Bangladesh).

[ref-6] Bhaskar RK, Sah MN, Gaurav K, Bhaskar SC, Singh R, Yadav MK, Ojha S (2016). Prevalence and correlates of tobacco use among adolescents in the schools of Kalaiya, Nepal: a cross-sectional questionnaire-based study. Tobacco Induced Diseases.

[ref-7] Cavazos-Rehg PA, Krauss M, Sowles S, Spitznagel EL, Grucza R, Chaloupka FJ, Bierut LJ (2016). Multiple levels of influence that impact youth tobacco use. Tobacco Regulatory Science.

[ref-8] Creamer MR, Delk J, Case K, Perry CL, Harrell MB (2018). Positive outcome expectations and tobacco product use behaviors in youth. Substance Use & Misuse.

[ref-9] Daniel WW (2009). Biostatistics.

[ref-10] Elshatarat RA, Yacoub MI, Khraim FM, Saleh ZT, Afaneh TR (2016). Self-efficacy in treating tobacco use: a review article. Proceedings of Singapore Healthcare.

[ref-11] Ernst M, Pine DS, Hardin M (2006). Triadic model of the neurobiology of motivated behaviour in adolescence. Psychological Medicine.

[ref-12] Glanz K, Rimer BK, Viswanath K (2015). Health behavior.

[ref-13] Global Youth Tobacco Survey Collaborative Group (2012). https://www.datafirst.uct.ac.za/dataportal/index.php/catalog/645/download/9102.

[ref-14] Haddock CK, Weg MV, DeBon M, Klesges RC, Talcott GW, Lando H, Peterson A (2001). Evidence that smokeless tobacco use is a gateway for smoking initiation in young adult males. Preventive Medicine.

[ref-15] Huang J, Chaloupka FJ (2012). The impact of the 2009 federal tobacco excise tax increase on youth tobacco use.

[ref-16] Huque R, Zaman MM, Huq SM, Sinha DN (2017). Smokeless tobacco and public health in Bangladesh. Indian Journal of Public Health.

[ref-17] Hussain A, Zaheer S, Shafique K (2017). Individual, social and environmental determinants of smokeless tobacco and betel quid use amongst adolescents of Karachi: a school-based cross-sectional survey. BMC Public Health.

[ref-18] IARC (2007). Smokeless tobacco and some tobacco-specific N-nitrosamines. IARC Monographs on the Evaluation of Carcinogenic Risks to Humans.

[ref-19] Islam N, AlKhateeb M (1995). Challenges and opportunities for tobacco control in the Islamic countries-a case-study from Bangladesh. Eastern Mediterranean Health Journal.

[ref-20] Islam SMS, Mainuddin AKM, Bhuiyan FA, Chowdhury KN (2016). Prevalence of tobacco use and its contributing factors among adolescents in Bangladesh: results from a population-based study. South Asian Journal of Cancer.

[ref-21] Kaduri P, Kitua H, Mbatia J, Kitua AY, Mbwambo J (2008). Smokeless tobacco use among adolescents in Ilala Municipality, Tanzania. Tanzania Journal of Health Research.

[ref-22] Kautilya VD, Sathish KV, Hegde SP (2015). Study of substance abuse among street children near Bangalore Medical College, Bangalore. Indian Journal of Forensic and Community Medicine.

[ref-23] Khariwala SS, Carmella SG, Stepanov I, Fernandes P, Lassig AA, Yueh B, Hatsukami D, Hecht SS (2013). Elevated levels of 1-hydroxypyrene and N′-nitrosonornicotine in smokers with head and neck cancer: a matched control study. Head & Neck.

[ref-24] Levy DT, Mays D, Boyle RG, Tam J, Chaloupka FJ (2017). The effect of tobacco control policies on US smokeless tobacco use: a structured review. Nicotine & Tobacco Research.

[ref-25] Liu ST, Nemeth JM, Klein EG, Ferketich AK, Kwan M, Wewers ME (2015). Risk perceptions of smokeless tobacco among adolescent and adult users and nonusers. Journal of Health Communication.

[ref-26] Mantler T (2013). A systematic review of smoking youths’ perceptions of addiction and health risks associated with smoking: utilizing the framework of the health belief model. Addiction Research & Theory.

[ref-27] Mia MN, Hanifi SMA, Rahman MS, Sultana A, Hoque S, Bhuiya A (2017). Prevalence, pattern and sociodemographic differentials in smokeless tobacco consumption in Bangladesh: evidence from a population-based cross-sectional study in Chakaria. BMJ Open.

[ref-28] Muttapppallymyalil J, Sreedharan J, Divakaran B (2010). Smokeless tobacco consumption among schoolchildren. Indian Journal of Cancer.

[ref-29] Mutti S, Reid J, Gupta P, Pednekar M, Dhumal G, Nargis N, Hussain A, Hammond D (2016). Patterns of use and perceptions of harm of smokeless tobacco in Navi Mumbai, India and Dhaka, Bangladesh. Indian Journal of Community Medicine.

[ref-30] Nargis N, Hussain AK, Fong GT (2014). Smokeless tobacco product prices and taxation in Bangladesh: findings from the International Tobacco Control Survey. Indian Journal of Cancer.

[ref-31] National Cancer Institute and Centers for Disease Control and Prevention (2014). Smokeless tobacco and public health: a global perspective.

[ref-32] Nemeth J, Liu S, Klein E, Ferketich A, Kwan M, Wewers M (2012). Factors influencing smokeless tobacco use in rural Ohio Appalachia. Journal of Community Health.

[ref-33] Perfetti TA, Rodgman A (2012). The chemical components of tobacco and tobacco smoke.

[ref-34] Renuka P, Pushpanjali K (2014). Effectiveness of health belief model in motivating for tobacco cessation and to improving knowledge, attitude and behaviour of tobacco users. Cancer and Oncology Research.

[ref-35] Sansone G (2014). Acceptability of female smoking and smokeless tobacco use in Bangladesh and India.

[ref-36] Siddiqi K, Shah S, Abbas SM, Vidyasagaran A, Jawad M, Dogar O, Sheikh A (2015). Global burden of disease due to smokeless tobacco consumption in adults: analysis of data from 113 countries. BMC Medicine.

[ref-37] Sidhu AK, Sussman S, Tewari A, Bassi S, Arora M (2016). Project EX-India: a classroom-based tobacco use prevention and cessation intervention program. Addictive Behaviours.

[ref-38] Sinha DN, Palipudi KM, Jones CK, Khadka BB, Silva PD, Mumthaz M, Shein NNN, Gyeltshen T, Nahar K, Asma S, Kyaing NN (2014). Levels and trends of smokeless tobacco use among youth in countries of the World Health Organization South-East Asia Region. Indian Journal of Cancer.

[ref-39] Warren CW, Lea V, Lee J, Jones NR, Asma S, McKenna M (2009). Change in tobacco use among 13–15-year olds between 1999 and 2008: findings from the Global Youth Tobacco Survey. Global Health Promotion.

[ref-40] World Health Organization (WHO) (1998). World health report 1998: life in the 21st Century, a vision for all.

[ref-41] World Health Organisation (WHO) (2013). WHO — Bangladesh –National tobacco control law amended. http://www.who.int/fctc/implementation/news/news_ban/en/.

[ref-42] World Health Organization (WHO) (2015). Global Youth Tobacco Survey (GYTS), Bangladesh Report, 2013.

[ref-43] World Health Organization (WHO), Regional Office for South-East Asia (2009). Global adult tobacco survey: Bangladesh report 2009.

