# Supplementary material for: Smokeless tobacco use: pattern of use, knowledge and perceptions among rural Bangladeshi adolescents
Source: PeerJ. 2018 Aug 21;6:e5463. doi: 10.7717/peerj.5463 (PMC6108312; doi:10.7717/peerj.5463)
Supplement: Supplemental Information 1 — Data collection Instrument [file peerj-06-5463-s002.docx]

Risk and Prevalence of Oral Cancer and Oral Leukoplakia of Smokeless tobacco use in Bangladesh

Survey Questionnaire for School:

Instructions:

- Please read each question carefully before answering it.
- Choose the answer that best describes what you believe and feel to be correct. We want to know what you think, whatever you believe will be the correct answer
- Choose only **one** answer for each question unless asked to choose more than one.
- On the answer sheet, locate the number that corresponds to your answer and fill it in completely with the pencil that was provided to you.
- If you have to change your answer, don’t worry; just erase it completely, without leaving marks.
- Do not put your name on the survey. We do not want to know who answered the questions
- The answers you give will not be shared with your teacher or your parents. The information will be used by the researcher (Dr Zahid) for his University degree in England.
- If you do not want to fill in the questionnaire, you do not have to.

## **Introduction:**

Thank you for participating in this survey. Before you start, please read the following information that will help you to answer the questions:

- ***Some of the questions will ask you about smokeless tobacco, which is tobacco that is not smoked, but is chewed, held in the mouth, or sniffed through the nose. Such as: Zarda, Sadapata, Pan Masala, Gutka, Gul; etc.***

About You:

**A1. Are you?**

- Male
- Female

**A2. How old are you?** __________Years old

**A3. What is your father’s job if he is working? ____________________________________________.**

**A4. What is your mother’s job if she is working? __________________________________________.**

Smokeless Tobacco Habits:

**B1. Have you ever used smokeless tobacco, such as Pan with Jarda, Gul, Pan Masala etc, even just a small amount?**

- Yes (If yes, then what brand or type of smokeless tobacco you used ___________________________)
- No

**[If you tried smokeless tobacco, then answer the next questions. Otherwise, go to B15.]**

**B2. How old were you when you first tried using smokeless tobacco?**

- I have never tried using smokeless tobacco
- 7 years old or younger
- 8 or 9 years old
- 10 or 11 years old
- 12 or 13 years old
- 14 or 15 years old

**B3. During the past 30 days, on how many days did you use smokeless tobacco?**

- 0 days
- 1 or 2 days
- 3 to 5 days
- 6 to 9 days
- 10 to 19 days
- 20 to 29 days
- All 30 days.

**B4. How many times did you usually use smokeless tobacco per day, in past 30 days?**

- I did not use smokeless tobacco during the past 30 days
- Less than once per day
- Once per day
- 2 to 5 times per day
- 6 to 10 times per day
- 11 to 20 times per day
- More than 20 times per day.

**B5. Do you ever use smokeless tobacco or feel like using smokeless tobacco first thing in the morning?**

- No, I don’t use or feel like using smokeless tobacco first thing in the morning
- Yes, I sometimes use or feel like using smokeless tobacco first thing in the morning
- Yes, I always use or feel like using smokeless tobacco first thing in the morning.

**B6.How soon after you use smokeless tobacco, do you start to feel a strong desire to use it again that is hard to ignore?**

- I never feel a strong desire to use it again after using smokeless tobacco
- Within 60 minutes
- 1 to 2 hours
- More than 2 hours to 4 hours
- More than 4 hours but less than one full day
- 1 to 3 days
- 4 days or more.

**B7. Why do you use smokeless tobacco? (You can have more than one answer for this question)**

- Taste
- Smell
- Pleasure
- To feel better/good/Happy
- Because my friend is using it
- Don’t Know.
- Other reason (Please Specify)**_____________________________**

**B8. Do you want to stop using smokeless tobacco now?**

- I don’t use smokeless tobacco now
- Yes
- No

**B9. During the past 12 months, did you ever try to stop using smokeless tobacco?**

- I did not use smokeless tobacco during the past 12 months
- I tried, but not successful
- Yes
- No

**B10**. **If you have tried to stop, but not successful, Why? _________________________________________**

**B11. Have you ever received help or advice to help you stop using smokeless tobacco? (If necessary, you can give more than one answer)**

- Yes, from a program or professional
- Yes, from a friend
- Yes, from a family member
- No

**B12. The last time you used smokeless tobacco during the past 30 days, how did you get it? (If necessary, you can give more than one answer)**

- I did not use smokeless tobacco during the past 30 days
- I bought it in a store or shop in the school canteen
- I bought it from a street vendor outside the school gate
- I got it from someone else
- I bought it from a store near to my house
- i bought it from a store on the way to school
- I got it some other way.- if you are willing to state how, please do - **_____________________________**

**B13. During the past 30 days, did anyone refuse to sell you smokeless tobacco because of your age?**

- I did not try to buy smokeless tobacco during the past 30 days
- Yes, someone refused to sell me smokeless tobacco because of my age
- No, my age did not keep me from buying smokeless tobacco.

**B14. During the past 30 days, did you see any health warnings on smokeless tobacco packages?**

- Yes, but I didn’t think much of them
- Yes, and they led me to think about quitting smokeless tobacco or not starting smokeless tobacco
- No .

**B15. If one of your best friends offered you smokeless tobacco, would you use it?**

- Definitely not
- Probably not
- Probably yes
- Definitely yes

**B16. Once someone has started using smokeless tobacco, do you think it would be difficult for them to quit?**

- Definitely not
- Probably not
- Probably yes
- Definitely yes

Health effects of Smokeless tobacco:

**C1. Do you think smokeless tobacco use is:**

- Good for your health
- Neither good nor bad for your health
- Not good for your health
- Don't Know

**C2. Are there benefits of smokeless tobacco to your body and health?**

- Yes, Please name them______________________________________________________________
- No

**C3. Does smokeless tobacco cause less harm to your health compare to smoking tobacco?**

- Yes
- No
- Don’t Know.

**C4. Does smokeless tobacco cause white patches in the mouth?**

- Yes
- No
- Don’t Know.

**C5. Can smokeless tobacco cause oral cancer?**

- Yes
- No
- Don’t Know

**C6. Does smokeless tobacco cause Gum diseases (*Gum disease is an infection of the gum that surround and support your teeth*)?**

- Yes
- No
- Don’t Know

**C7. Does smokeless tobacco cause heart disease?**

- Yes
- No
- Don’t Know.

**C8. Does smokeless tobacco contains nicotine?*(Nicotine is a chemical that is present in cigarettes that makes people become addicted)?***

- Yes
- No
- Don’t Know.
