# Supplementary material for: Smokeless tobacco use: pattern of use, knowledge and perceptions among rural Bangladeshi adolescents
Source: PeerJ. 2018 Aug 21;6:e5463. doi: 10.7717/peerj.5463 (PMC6108312; doi:10.7717/peerj.5463)
Supplement: Supplemental Information 2 [file peerj-06-5463-s003.docx]

# Model Validation:

| **Table . Model diagnostics** | | | | |
| --- | --- | --- | --- | --- |
| df | Likelihood Chi square |  | Likelihood P value | Model Accuracy |
| 6 | 41.4 |  | 2 x 10 ^-7^ | 82% |

Results show that the likelihood test result was significant which indicates that our model fits the data better than the Null model (model with no predictors) where the p value for the likelihood ratio test was less than 0.05.

The model correctly classified 82% of the dependent variable which is considered fairly accurate.

**Fit measures**

| **Pseudo R^2^** | **Value.** |
| --- | --- |
| McFadden | 0.166 |
| Adjusted McFadden | 0.102 |
| Cox Snell | 0.051 |
| Nagelkerke | 0.189 |
| AIC | 221.198 |
| Corrected AIC | 221.341 |

While no exact equivalent to the R2 of linear regression exists, the McFadden R2 index can be used to assess the model fit. Values between 0.2 and 0.4 indicate excellent model fit. Our model has an R2 of 0.166. However, these measures cannot be used alone to judge the model.


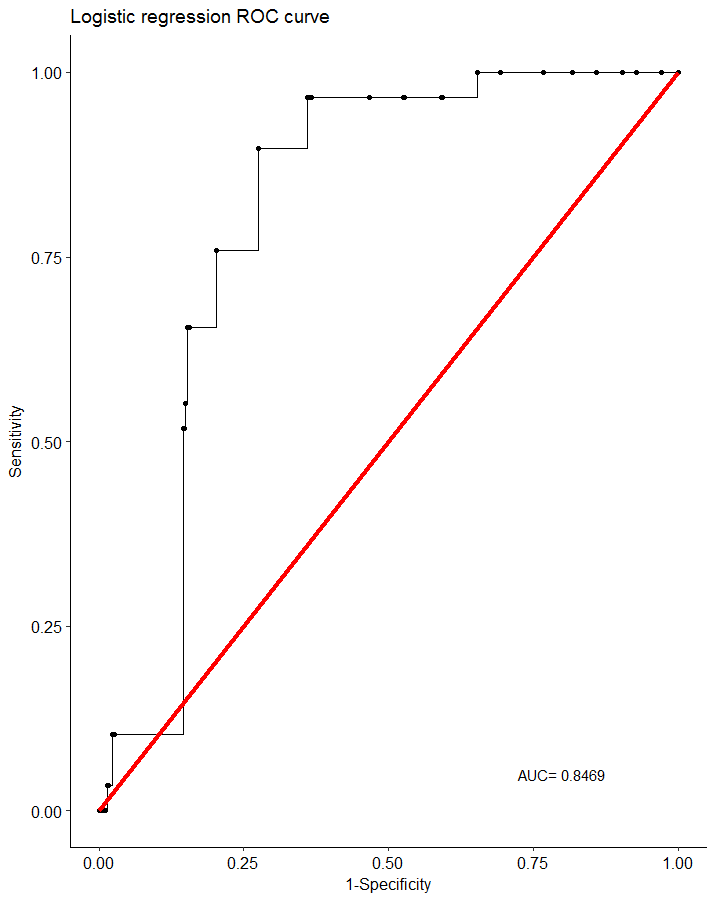


As previously mentioned, the model correctly classified 82% of the data. The ROC curve shows that the AUC is 84.69% which indicates a good predictive power (>0.8).

As a rule of thumb, a model with good predictive ability should have an AUC closer to 1 (1 is ideal) than to 0.5.


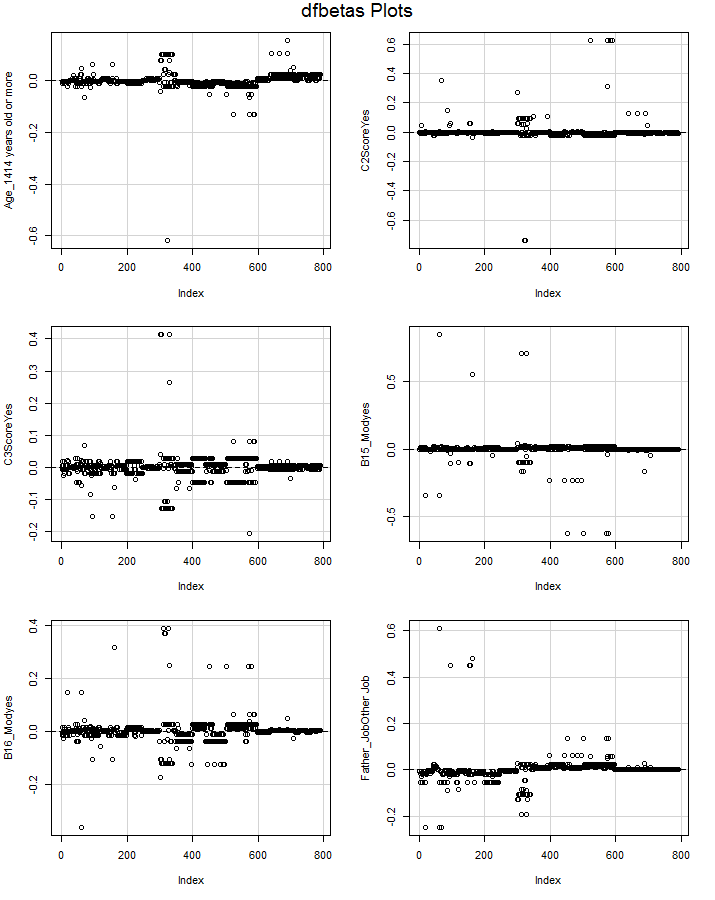
Deviance residuals also indicate that the model was a good fit for the data where the coefficients did not change significantly with the removal of any of the data points.
